# Trade-offs in sensitivity and sampling depth in bimodal atomic force microscopy and comparison to the trimodal case

**DOI:** 10.3762/bjnano.5.125

**Published:** 2014-07-24

**Authors:** Babak Eslami, Daniel Ebeling, Santiago D Solares

**Affiliations:** 1 Department of Mechanical Engineering, University of Maryland, College Park, MD 20742, USA; 2 present address: Institute of Applied Physics, Justus Liebig University of Giessen, 35392 Giessen, Germany

**Keywords:** amplitude modulation, bimodal, multifrequency atomic force microscopy, indentation depth modulation, Nafion, open loop, proton exchange membranes, trimodal

## Abstract

This paper presents experiments on Nafion^®^ proton exchange membranes and numerical simulations illustrating the trade-offs between the optimization of compositional contrast and the modulation of tip indentation depth in bimodal atomic force microscopy (AFM). We focus on the original bimodal AFM method, which uses amplitude modulation to acquire the topography through the first cantilever eigenmode, and drives a higher eigenmode in open-loop to perform compositional mapping. This method is attractive due to its relative simplicity, robustness and commercial availability. We show that this technique offers the capability to modulate tip indentation depth, in addition to providing sample topography and material property contrast, although there are important competing effects between the optimization of sensitivity and the control of indentation depth, both of which strongly influence the contrast quality. Furthermore, we demonstrate that the two eigenmodes can be highly coupled in practice, especially when highly repulsive imaging conditions are used. Finally, we also offer a comparison with a previously reported trimodal AFM method, where the above competing effects are minimized.

## Introduction

Since its invention in the early 1980s [1], atomic force microscopy (AFM) has become one of the most widely used characterization tools in nanotechnology and a wide range of imaging modes is now available, each with its own capabilities and applications. Among them, a family of techniques known as multifrequency AFM [2–11] has expanded considerably since the introduction of the first bimodal method by Rodriguez and Garcia in 2004 [12]. In multifrequency AFM the cantilever probe is driven simultaneously at more than one frequency, with the objective of creating additional channels of information in order to provide a more complete picture of the sample morphology and properties [2].

In the original method of Garcia and coworkers [12–13] the first eigenmode of the cantilever is driven using the amplitude modulation scheme (AM-AFM [14]) while a higher eigenmode is simultaneously driven at or near its resonance frequency with constant amplitude and frequency (i.e., in “open loop”) in order to track its phase with respect to the excitation signal. Since the higher eigenmode is not directly subject to the amplitude modulation control loop that governs the acquisition of the topography, the user has freedom in selecting its operating parameters, thus allowing it to explore a wider range of tip–sample interactions. Additionally, since its amplitude is generally smaller than that of the fundamental mode, it can be made more sensitive to compositional contrast, as previously discussed by Rodriguez and Garcia [12]. The two eigenmodes can also be driven using the frequency modulation scheme (FM-AFM [4,15–17]), and it is also possible to simultaneously drive more than two eigenmodes. In a recently introduced trimodal method, two eigenmodes are used for topographical imaging and compositional mapping, respectively, and a third one is used to modulate the tip indentation depth during imaging [9]. The modulation of the indentation depth is accomplished through changes in the amplitude of the highest driven eigenmode, which has the highest dynamic force constant (the higher stiffness of higher eigenmodes has also been advantageous in subsurface imaging applications in contact resonance AFM [18]). In this paper we show that indentation depth modulation can also be accomplished when using bimodal AFM, although without the flexibility to independently optimize the sensitivity of the compositional mapping process. We discuss the trade-offs involved and provide an illustration of the dynamics complexities, including strong eigenmode coupling in some cases. Finally, we also offer a comparison to the trimodal method [9]. Note that in this paper we use the word sensitivity to *qualitatively* describe the ability of an eigenmode observable (e.g., phase shift) to detect small changes in the tip–sample forces, which in turn are governed by the surface properties. Since much of the discussion is based on the cantilever dynamics, the term can also be understood as the ability of a given cantilever eigenmode to be perturbed by small changes in the external forces when it is oscillating under the specified parameters. Our discussion and conclusions are based on the ideal case where noise is not a limitation.

## Results and Discussion

### Repulsive vs attractive imaging

**Figure 1:**
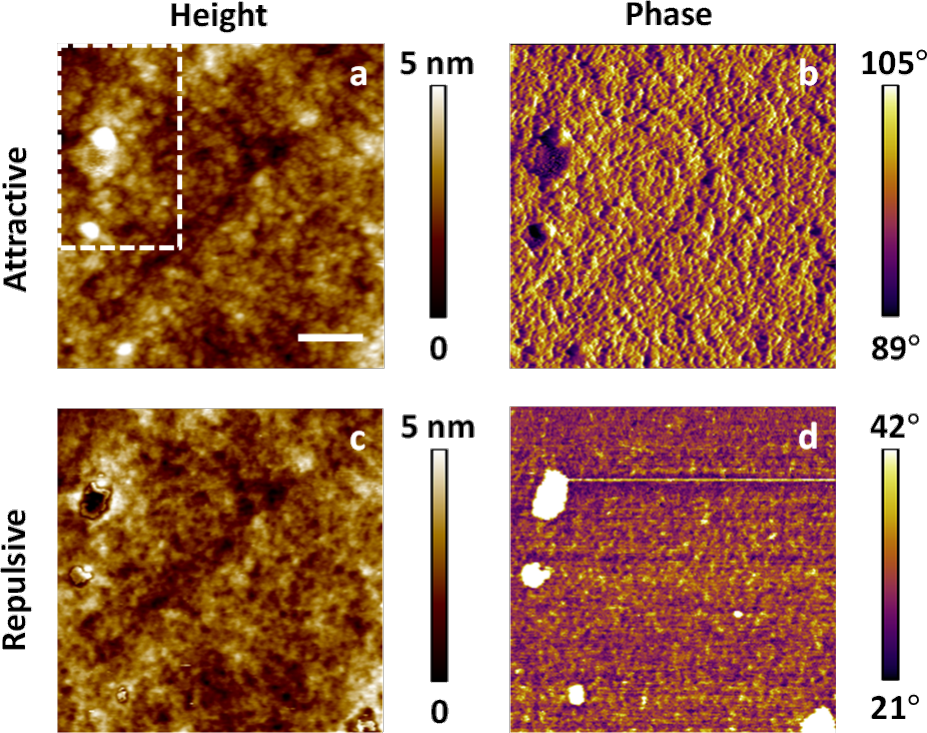
(a) and (b) topography and phase images, respectively, of a Nafion^®^ membrane acquired in the attractive regime; (c) and (d) corresponding images acquired in the repulsive regime. The scale bar is 100 nm. The morphology of the region in the dashed rectangle in (a) is discussed below in Figure 6. The free oscillation amplitude in both cases was 17 nm, with an amplitude setpoint of 80% for attractive regime imaging and 50% for repulsive regime imaging.

In general, nanoscale surfaces can be imaged with AFM in either the attractive or repulsive imaging regime [14]. In the attractive regime the overall interaction between the cantilever tip and the sample surface is not affected by forces originating from physical contact. Instead, changes in the non-contact tip–sample interactions, which include van der Waals, electrostatic and magnetic forces, establish the basis for mapping sample topography and properties. In fact, the first bimodal AFM implementation of Rodriguez and Garcia was for imaging in the attractive regime [12–13]. In the repulsive imaging regime the cantilever tip intermittently impacts the sample and thus the images are governed by contact forces that are a consequence of elastic, plastic, viscous or adhesive surface behaviors, in addition to the noncontact forces. Figure 1 provides an example of single-mode attractive and repulsive images of a Nafion^®^ fuel cell membrane (these images were acquired by using the standard amplitude modulation method [14]). A difference between the two images can be seen in terms of contrast inversion, feature sizes, shapes and patterns, which have been previously attributed by others to the competing effects of membrane functionality and contact mechanics on the cantilever response [19].

**Figure 2:**
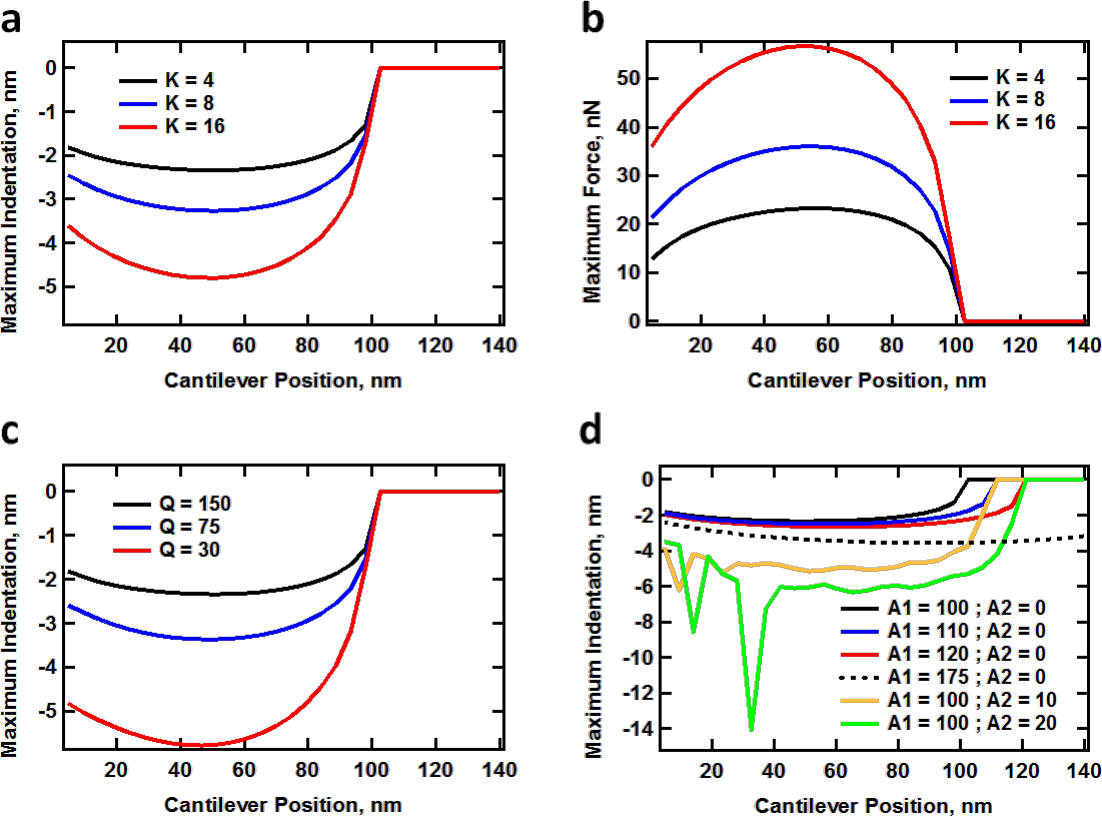
Simulations of maximum indentation and peak force (see section Methods below for details on the numerical simulations of the cantilever dynamics as well as the tip–sample force model used): (a) maximum indentation depth vs cantilever force constant; (b) peak forces corresponding to (a); (c) maximum indentation vs cantilever quality factor, *Q* (unrealistically low values of *Q* were chosen to illustrate the effect of high damping); (d) maximum indentation vs first and second eigenmode free amplitudes. The first eigenmode free amplitude in these simulations was 100 nm, unless otherwise indicated. The cantilever and force model parameters are provided in section Methods. The irregular behavior of the indentation for the lowest two traces at small cantilever–sample separations in (d) is a consequence of the non-steady state behavior of multi-eigenmode oscillations [9,22].

In cases in which the mechanics of the subsurface are of interest, it is necessary to operate the AFM in a way that the indentation depth can be controlled. This could be achieved in single-mode operation in a number of ways, including the use of cantilevers with different spring constants (see Figure 2a and Figure 2b), the use of cantilevers with different quality factors (Figure 2c), changes in the amplitude setpoint, or changes in the free oscillation amplitude. The first option is not practical since it requires a cantilever changeover. The second option is feasible using the *Q*-control method but requires additional electronics [20–21]. The third option does not work indefinitely since indentation and peak forces vary in a non-monotonic fashion as the cantilever is lowered towards the surface, as illustrated by all plots in Figure 2 [9]. The fourth option is also relatively limited in the additional indentation depth that can be accomplished as shown in Figure 2d (black, blue, red and dotted black traces).

### Indentation depth modulation with bimodal AFM

Figure 2d shows that the second eigenmode is much more effective in accomplishing additional indentation depth than the fundamental eigenmode for the same change in amplitude (note how a 10 nm increase in the second mode amplitude is significantly more effective in increasing the indentation depth under the conditions illustrated than a 75 nm increase in the first mode amplitude), although the level of penetration into the sample can exhibit non-smooth behavior due to the non-steady state behavior of multifrequency oscillations [9,22], especially as the cantilever is brought very close to the surface (in these simulations the curves become smoother if one considers a larger number of taps for every cantilever height in the construction of the graphs). As previously reported, the greater indentation capability of higher eigenmodes with respect to the fundamental mode can be understood by inspecting the dimensionless equation of motion of a damped harmonic oscillator [9].

[1]
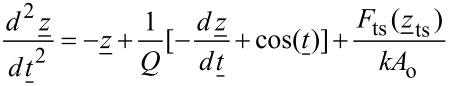


where *A*_o_ is the free amplitude, *z* = *z*(*t*)/*A*_o_ is the dimensionless tip position with respect to the cantilever base, *z*_ts_ = *z*_ts_/*A*_o_ is the dimensionless tip–sample distance (*z*_ts_ = *z* + *z*_eq_, where *z*_eq_ is the position of the cantilever above the sample), *t* = ω_o_*t* is the dimensionless time, *k* is the cantilever force constant and *F*_ts_ is the tip–sample interaction force. We have made the substitution *A* ≈ *A*_o_ = *F*_o_*Q*/*k* [14], where *F*_o_ is the amplitude of the excitation force, and we have combined the damping and excitation terms with the factor *1/Q.* The last term on the right hand side indicates that the tip–sample forces are normalized by the product of the force constant times the free amplitude. Thus, the external forces influence the dynamics more or less when the product *kA*_o_ becomes smaller or larger, respectively. As the product *kA*_o_ decreases, the oscillator is more easily perturbed by the tip–sample forces (i.e., it is more sensitive to external forces), whereas the perturbations are less significant when this product increases. Thus, if the objective is to obtain the greatest gain in controlling indentation for a given cantilever, one should choose for this purpose the highest available eigenmode, which has the highest dynamic force constant and thus the largest product *kA*_o_ for a given value of *A*_o_ (the dynamic force constants of the cantilever eigenmodes increase with the square of their eigenfrequency – see Table 1 in [2]). However, one must be mindful that increasing indentation in this manner comes with a loss in sensitivity. In other words, since greater indentation is being accomplished by driving the cantilever in a way in which it is less able to be perturbed by the tip–sample forces (greater repulsive forces are required to perturb it, which leads to greater penetration into the repulsive part of the tip–sample potential), it can also be more difficult to detect small changes in the behavior of the tip–sample forces, which are related to the sample properties. The choice depends on what the user's highest priority is – indentation depth or sensitivity in compositional mapping.

**Figure 3:**
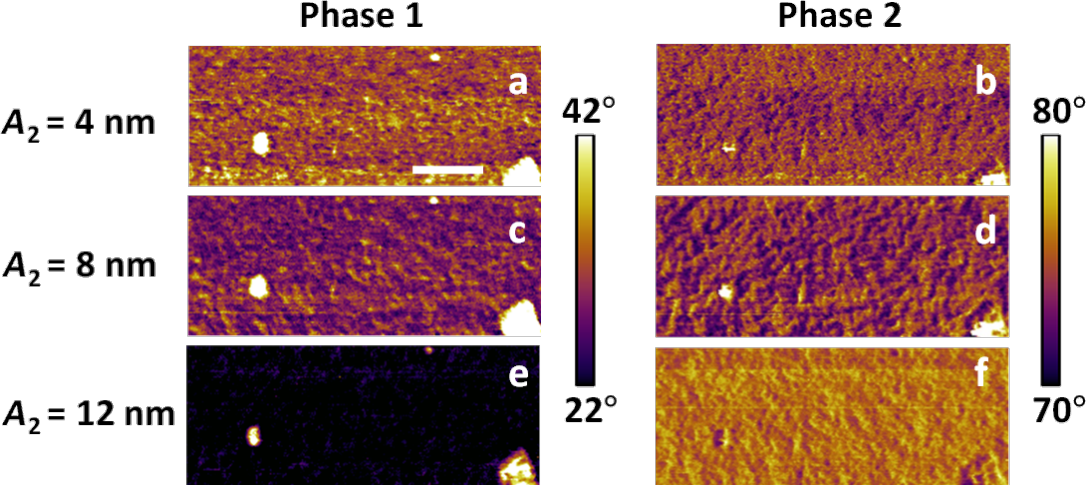
Bimodal experiments with varying second eigenmode amplitude for a Nafion^®^ membrane (the images correspond to the lower portion of those shown in Figure 1). The left and right columns provide, respectively, the phase of the first and second eigenmode. Each row is a separate experiment using the second mode free amplitude indicated on the left. The second amplitude was increased in the downward direction to increase indentation, according to Figure 2d. The first eigenmode free amplitude was 17 nm with a setpoint of 45%. The scale bar is 100 nm.

Since each eigenmode is governed by an equation similar to Equation 1 (except that there are additional cosine driving force terms, one for each driven eigenmode) and the tip–sample forces are the same for all equations, the various eigenmodes are coupled and the degree of coupling becomes more noticeable in the dynamics as the higher eigenmode amplitude increases. This is illustrated in Figure 3, in which each row represents a separate experiment for a given free oscillation amplitude of the second eigenmode. The scale bar for each respective phase (first or second) is the same for all experiments. One can easily see that as the second eigenmode amplitude increases and the indentation increases according to Figure 2d, the first eigenmode phase changes drastically indicating a more repulsive interaction (the values decrease for each successive row, which corresponds to higher and higher second mode amplitudes). The first eigenmode has the lowest dynamic force constant, so it is more easily perturbed by the dynamics of the second eigenmode. In contrast, the phase values of the second eigenmode increase for successive rows (which would indicate a less repulsive regime since the values are closer to the 90° phase shift of the unperturbed oscillator).

**Figure 4:**
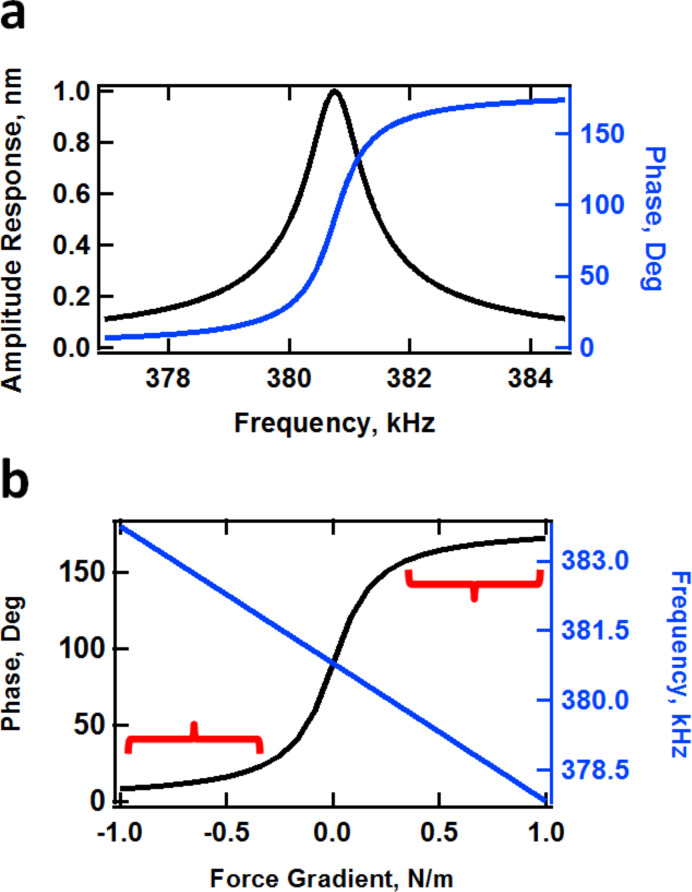
Illustration of the ideal response of a harmonic oscillator [22]. (a) Amplitude and phase vs excitation frequency (at the resonance frequency the phase is 90 degrees); (b) phase and effective frequency shift vs external force gradient (at zero force gradient the phase is 90 degrees and the frequency is equal to the resonance frequency). In general, the force gradient becomes more negative (the force curve becomes steeper in the repulsive region) as the tip–sample indentation increases, leading to lower phase values. The red brackets in (b) indicate regions of low contrast, where the phase response is relatively flat with respect to changes in the force gradient. Measurements under these conditions lead to lower quality contrast in the images.

**Figure 5:**
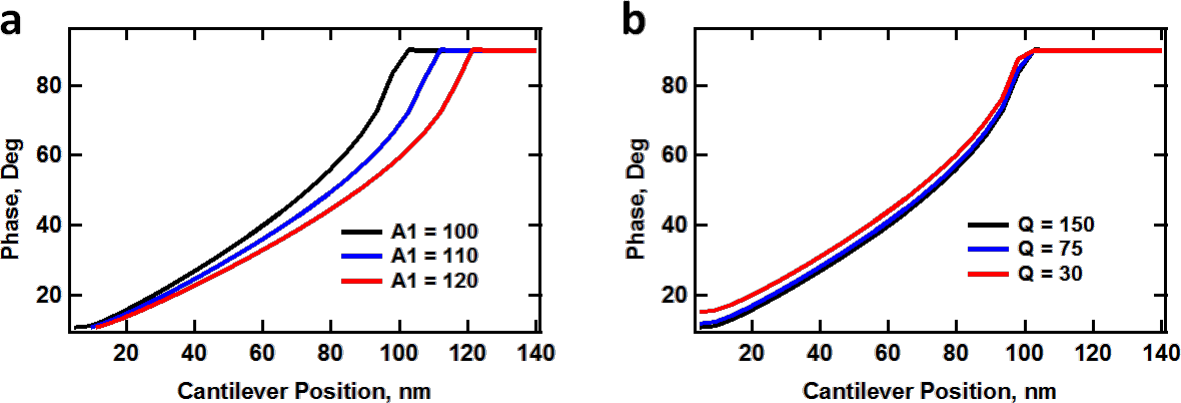
Simulated behavior of the first eigenmode phase as a function of free amplitude (a) and cantilever quality factor (b). See Figure 2 for the corresponding behavior of the indentation.

To understand the above result we have to consider the two competing effects that are at play. As the second mode amplitude increases, this eigenmode becomes less sensitive (more difficult to perturb), which leads to larger phase values (closer to the neutral phase value of 90°, which is observed when no tip–sample forces are present and the eigenmode is driven at the natural frequency). However, since the relationship between the tip–sample forces and the phase and amplitude is very difficult to establish [23], and the amplitude is not constant for successive rows in the experiments of Figure 3, increasing phase values are not an unambiguous criterion that one can use to conclude that the repulsive forces for this eigenmode are becoming smaller. Additionally, the decrease in sensitivity leads to greater indentation and thus to larger and steeper tip–sample forces, which would have the opposite effect of lowering the phase further away from 90° (see Figure 4b). Depending on the sample and the tip, which govern the behavior of the forces as a function of tip position and velocity, one of these two effects will dominate. In this particular case, the loss in sensitivity dominates and the phase values increase (see also reference [24] for a similar type of experiment on a polystyrene–polybutadiene diblock copolymer). This result may or may not be desirable, depending on what information is sought (surface contrast, high-indentation surface morphology, etc.). Furthermore, as indentation is modulated through free amplitude changes, it is important to consider whether the phase response is in the high or low contrast region (the low contrast regions are those where the phase response becomes nearly flat with respect to changes in the external force gradient, as indicated in Figure 4b). Although the tip–sample force model is not generally available during an experiment, it is also important to consider at least conceptually whether the changes in imaging conditions lead to more or less sensitive phase response for a given type of sample. Figure 5a shows an illustration of the (simulated) phase behavior for the standard linear solid model used here (see section Methods for further details). Clearly the phase response as a function of the cantilever position becomes flatter with respect to the cantilever position above the surface when the first eigenmode amplitude is increased, whereas Figure 5b shows that the phase curve slope behaves similarly for different values of the quality factor (although the cantilever quality factor cannot be arbitrarily changed, the *effective* quality factor can vary significantly during the measurement due to the dissipative tip–sample interactions, which also cause a decrease in amplitude that leads to additional changes in eigenmode sensitivity [22]). In general, steeper responses of the imaging variables are desired with respect to changes in the imaging conditions (e.g., phase vs cantilever height, or equivalently, phase vs amplitude setpoint, in the case of Figure 5).

**Figure 6:**
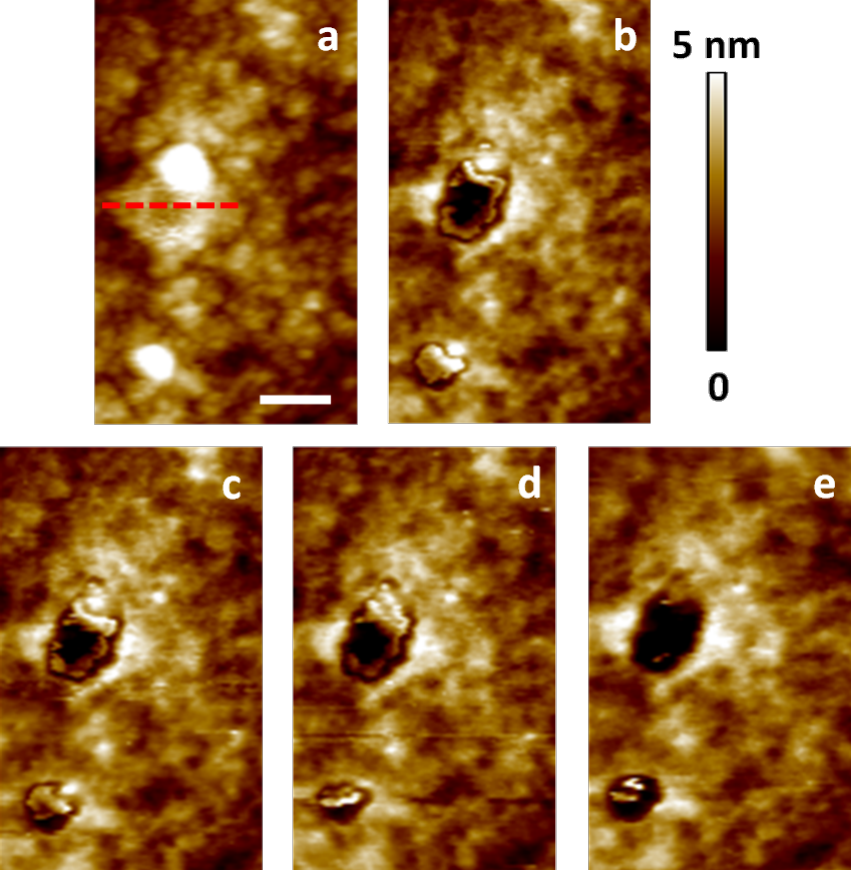
Morphology change of the topographical feature highlighted in Figure 1a for different imaging conditions. (a) and (b) show results taken from the images shown in Figure 1a and 1c, respectively. The panels (c–e) show results acquired during the three experiments shown in Figure 3 with increasing indentation (from the top row to the bottom row in Figure 3). This behavior is initially reversible but becomes irreversible after repeated imaging under highly repulsive conditions. The scan line profiles along the dashed red line for (a), (b), (c) and (e) are provided in Figure 7. The scale bar is 50 nm.

**Figure 7:**
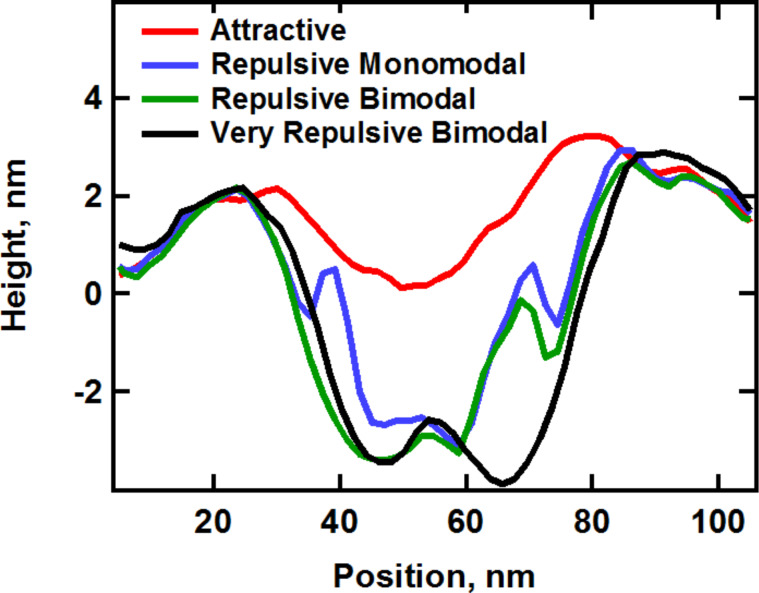
Scan line profiles along the dashed line indicated in Figure 6a for the images shown in Figure 6a (attractive), Figure 6b (repulsive monomodal), 6c Figure (repulsive bimodal) and Figure 6e (very repulsive bimodal).

An important consequence of the phenomena discussed above is that when a user ‘optimizes’ the imaging conditions in bimodal AFM to obtain the topography with the first eigenmode and to carry out compositional contrast with the higher eigenmode, changes in the higher mode amplitude lead not only to changes in the contrast sensitivity but also to changes in what region of the sample is actually being sampled (sampling region here refers to the volume of material between the surface skin and the lowest point reached by the tip during maximum indentation). Thus, images with drastically different parameters are not necessarily comparable to one another. Figure 6 illustrates the corresponding changes in the acquired topography for the feature highlighted in Figure 1a, for the single-mode attractive and repulsive imaging experiments (Figure 1) and the three sets of experiments shown in Figure 3, and Figure 7 gives the corresponding scan line profiles for four of the images along the dashed line indicated on Figure 6a. Clearly the topography and morphology can change significantly as more repulsive imaging conditions are sought, and these changes become more significant as the sample stiffness decreases (see also indentation-dependent measurements for a soft polymer film embedded with nanoparticles in [9]).

It may appear from the above discussion that the user has little control on what aspect or region of the sample is being characterized, but this is not necessarily the case. The observations presented here simply highlight the need for acquiring complementary information, especially through simulation, in order to carry out a sound interpretation of the results.

### Comparison to trimodal AFM

As already stated, in the AM-OL (amplitude modulation – open loop) bimodal method the ‘optimization’ of compositional contrast mapping and indentation depth modulation is accomplished with the same higher eigenmode, which does not allow the user to control them independently. Furthermore, these two objectives can compete against one another since greater indentation is accomplished by driving the cantilever in a way in which it is less likely to be perturbed by the tip–sample forces (i.e., in a way in which it is less sensitive). In contrast, in trimodal AFM each function is accomplished by a separate eigenmode: the fundamental mode is used for topographical acquisition, a higher mode is used for compositional mapping, and an even higher mode is used to modulate indentation [9]. Since the spectroscopy eigenmode can be optimized nearly independently through changes in its free amplitude, the non-responsive regions of the phase response (indicated by brackets in Figure 4b) can be avoided. Not only are all functions accomplished with separate modes, but the user is also able to space those modes as much as is desired. For example, since increasingly higher modes have increasingly larger force constants, one could use the second mode for compositional mapping while modulating indentation with the fourth or an even higher mode for a very stiff sample, whereas one would use the first three modes for a soft sample.

## Conclusion

We have examined through experiment and simulation the trade-offs between the optimization of compositional contrast and the modulation of tip–sample indentation for bimodal AFM combining amplitude modulation for topographical acquisition and open-loop drive for compositional mapping [12–13]. In general, it is possible to increase indentation in this mode of operation by increasing the amplitude of the higher mode, but this usually comes with a loss in sensitivity. We demonstrate that changes in sensitivity and indentation cannot be separated within this method. We have also illustrated the coupling of the two eigenmodes, whereby highly repulsive imaging conditions resulting from the choice of higher mode parameters can have a drastic effect in the response of the fundamental mode. Finally, we offer a comparison to a previously reported trimodal AFM method, whereby the modulation of indentation and the optimization of compositional contrast are carried out with separate eigenmodes, thus minimizing the above competing effects. Despite the limitations discussed, however, the AM-OL bimodal method remains an attractive alternative due to its relative simplicity, robustness and commercial availability. Furthermore, an in-depth knowledge of the dynamics and trade-offs involved can allow an experienced user to reach a favorable compromise between versatility and sensitivity.

## Methods

### Experimental

The experimental measurements were carried out on an Asylum Research (Santa Barbara, CA, USA) MFP3D-SA microscope, which is equipped with bimodal imaging modes. We used a Bruker (Santa Barbara, CA, USA) MPP-33120 cantilever with first two resonance frequencies at 45.99 and 284.39 kHz, respectively, fundamental force constant of 7.3 N/m and fundamental quality factor of 236. The amplitude of the first eigenmode was calibrated by using amplitude–distance curves and the amplitude of higher eigenmodes was estimated by using their respective optical sensitivity factors (see Table 1 in [2]).

The experimental sample consisted of the proton exchange membrane Nafion^®^ 115, purchased from Ion Power, Inc. (New Castle, DE, USA). The product was received in H^+^ form and no further treatment was performed, except for a routine cleaning procedure. Sections of the membrane of the size of 1 × 1 cm^2^ were rinsed with DI-water followed by 5 min of ultrasonication in DI-water and a second rinse step prior to equilibration in a closed container. During the experiments reported in this paper, the air in the AFM chamber was monitored to be at 23 °C and 17% relative humidity.

### Computational

For the numerical simulations the first three eigenmodes of the AFM cantilever were modeled by using individual equations of motion for each, coupled through the tip–sample interaction forces as in previous studies [9]. The first two eigenmodes were excited through respective sinusoidal tip forces of constant amplitude, with the drive frequencies matching the resonance frequencies. The equations of motion were integrated numerically and the amplitude and phase of each eigenmode were calculated using the customary in-phase (*I_i_*) and quadrature (*K_i_*) terms:

[2]
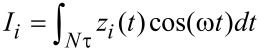


[3]
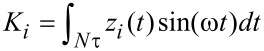


where *z_i_*(*t*) is the *i*-th eigenmode response in the time domain, *N* is the number of periods over which the phase and amplitude were averaged (we rounded *N* to the integer closest to 25 times the ratio of each eigenmode’s frequency to the fundamental frequency), ω is the excitation frequency, and τ is the *nominal* period of one oscillation. The amplitude and phase were calculated, respectively, as:

[4]
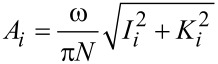


[5]
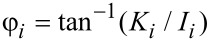


The repulsive tip–sample forces were accounted for through a standard linear solid (SLS) model [9] having force constants of 7.5 N/m for the two linear springs and a dashpot constant of 1 × 10^−5^ Ns/m. The long-range attractive interactions were included through the Hamaker equation [14] for a tip radius of curvature of 10 nm and a Hamaker constant of 2 × 10^−19^ J.
